# Sodium-Glucose Cotransporter-2 (SGLT-2) Inhibitors Use among Heart Failure Patients and the Role of Pharmacists in Early Initiation of Therapy

**DOI:** 10.3390/pharmacy11020058

**Published:** 2023-03-17

**Authors:** Mohammed Aldhaeefi, Brandon Beers, Jenny Shah, Saba Saeidi Rizi, Dhakrit Rungkitwattanakul, Oliver Nimoh, Victoria Frimpong, Jackie Gonzalez, Sanaa Belrhiti, Fatima Urooj, Deborah Williams

**Affiliations:** 1 Clinical and Administrative Pharmacy Sciences, College of Pharmacy, Howard University, Washington, DC 20059, USA; 2 Department of Pharmacy Services, Cone Health Alamance Regional Medical Center, Burlington, NC 27215, USA; 3 Department of Pharmacy Services, Howard University Hospital, Washington, DC 20060, USA; 4 College of Pharmacy, Howard University, Washington, DC 20059, USA; 5 Heart Centre, Howard University Hospital, Washington, DC 20060, USA; 6 College of Medicine, Howard University, Washington, DC 20059, USA

**Keywords:** heart failure, SGL-2 inhibitors, pharmacists, sodium-glucose cotransporter-2 inhibitors

## Abstract

Heart failure (HF) is a growing major public health and economic concern in the United States and worldwide. Heart failure mortality rates can be as high as 75% despite advances in therapies. HF is expected to be the fastest growing among all cardiovascular diseases, with HF-associated direct medical costs projected to nearly double over the next 10 years. Hospital admissions, re-admission, and medical cost are a huge burden to the healthcare system, and this is estimated to have increased gradually over the past decades despite the available advances in HF treatment and prevention. Many heart failure therapies have shown improvement in terms of mortality, morbidity, and symptomatic management. Guideline-directed medical therapy (GDMT) for heart failure has proven its ability to reduce morbidity and mortality by 66%. GDMT is recommended to be used among all HF patients when appropriate. In recent years, two new drug classes, angiotensin receptor-neprilysin inhibitor (ARNi) and sodium-glucose cotransporter-2 (SGLT-2) inhibitors, were approved by the United States Food and Drug Administration (US FDA) for the management of heart failure. The exact mechanism by which the SGLT-2 inhibitors attenuate the inflammatory process remains unclear. Several mechanisms have been suggested related to the cardiovascular benefit of SGLT-2 inhibitors, including a reduction in inflammation, improvement in natriuresis/diuresis, and promotion of the use of ketones as a secondary energy source. Clinical data showed that SGLT-2 inhibitors have morbidity and mortality benefits within 30 days of initiation. Studies have proven that clinical pharmacists practicing in HF inpatient and outpatient settings resulted in a reduction of HF hospitalization and an increase in the uptake of GDMT by initiating or up-titrating GDMT agents as well as providing patient education.

## 1. Introduction

Heart failure (HF) is a complicated clinical syndrome in which the body is not able to maintain adequate metabolic supply to organs and tissues due to structural and/or functional myocardial dysfunction [1]. In clinical practice, HF patients present with concurrent cardiovascular disease, such as atrial fibrillation and hypertension, or conditions that increase the risk of cardiovascular disease, such as diabetes [1,2]. HF is a growing major public health and economic concern [1]. Although we have made significant advances in the management of HF, 1-year mortality rates remain unacceptably high from 1998 to 2008 at ~30%, and 5-year mortality approaches 75% with uneven rates across states and by race [2,3]. Additionally, the majority of mortality reduction has been estimated to be in heart failure with reduced ejection fraction (HFrEF), leaving equally vulnerable populations of heart failure with midrange ejection fraction (HFmrEF) and heart failure with preserved ejection fraction (HFpEF) unchanged [3]. The burden of cardiovascular diseases (CVD) is expected to affect >40% of US adults by 2030. HF is anticipated to be the fastest growing contributor to underlying CVD, with HF-associated direct medical costs projected to nearly double over the next 10 years. Hospital admissions for heart failure are a huge burden to the healthcare system, and the volume of admissions is estimated to have increased gradually over the past decades despite the available advances in HF treatment and prevention [2].

The American College of Cardiology and the American Heart Association (ACC/AHA) established treatment guidelines for the management of heart failure, which were recently updated in 2022 from 2016 [4]. The ACC/AHA guidelines categorize heart failure as Stage A through D, nonetheless, the New York Heart Association (NYHA) classifies heart failure as class I-IV [4]. The management of stage A (“At Risk for Heart Failure”) and stage B (“Pre-Heart Failure”) patients is mainly focused on evidence-based management of other underlying comorbidities, such as hypertension, CVD, diabetes, obesity, and other, with an emphasis on lifestyle medication. However, the guidelines give strong recommendation to use select GDMT agents for compelling indications such as hypertension with ACEi/ARB, diabetes or CVD with SGLT-2 inhibitors, and recent myocardial infarction with EF ≤40% with beta blockers [4]. Over the past few years, two new drug classes—angiotensin receptor-nerpilysin inhibitor (ARNi) and sodium-glucose cotransporter-2 (SGLT-2) inhibitors—were approved by the United States Food and Drug Administration (US FDA) [5]. With further advanced stage C (“Symptomatic Heart Failure”) and stage D (“Advanced Stage Heart Failure”) patients, the ACC/AHA recommends the four pharmacotherapy pillars. Those pillars are ARNI, if tolerated, or ACEi/angiotensin receptor blockade (ARB) if ARNi resulted in intolerance; beta-blockers; mineralocorticoid receptor antagonists (MRA); and SGLT-2 inhibitors [4]. Heart Failure mortality and morbidity were lower when those pharmacotherapy pillars were utilized [4].

In recent decades, the pharmacological management of heart failure has become a true success story of modern medicine. Evidence from high-quality randomized controlled studies has been adopted as a basis of pharmacological management that improves both mortality and symptoms [5]. In fact, guideline-directed medical therapy (GDMT) when targeted doses were used for heart failure has proven its ability to reduce morbidity and mortality by 66%; however, utilization remains low [2]. Data from the CHAMP-HF registry showed that only between 10–25% of eligible patients received the target doses of GDMT agents, and only 1% of patients were simultaneously treated with target doses of all GDMT agents [6,7]. Barriers to utilizing GDMT in HF may include cost, clinical inertia, perception of siloed disease state management, preference for outpatient settings, or lack of up-to-date knowledge/information [7,8,9,10], lack of care coordination, poor transition of care planning, and patients’ adherence due to health literacy or lack of resources [7,8,9,10]. Caring for heart failure patients in a multidisciplinary care team model, including physician, pharmacist, nurse, and case manager, may help overcome some of these barriers [7,8,9,10].

Clinical Pharmacists practicing in HF care settings as part of the multidisciplinary team have been able to show a significant reduction in HF hospitalization and an increase in the uptake of GDMT by initiating or up-titrating GDMT agents as well as providing patient education [11,12]. Furthermore, clinical pharmacists interventions while caring for heart failure patients was shown to improve the cost effectiveness for healthcare systems [12,13]. Given the ongoing emerging evidence of SGLT-2 inhibitors related to the management of HF since their approval, our objectives for this review are to describe and explore the evidence that supports the use of Food and Drug Administration (FDA)-approved SGLT-2 inhibitors in HF patients, empagliflozin and dapagliflozin, and to discuss the role of pharmacists in the early initiation of SGLT-2 inhibitors as part of the GDMT in HF patients.

## 2. Mechanisms of Cardiovascular Benefits of SGLT-2 Inhibitors

Historically, SGLT-2 inhibitors were originally approved and utilized exclusively for glycemic control in patients with diabetes [14,15,16,17]. However, in recent years there has been an increase in the use of SGLT-2 inhibitors in patients with and without diabetes [18,19]. The first approved SGLT-2 inhibitor for heart failure by the US FDA was dapagliflozin in May 2017 [18]. Empagliflozin was granted FDA approval for adult patients with HFrEF in August 2021 [19]. When used for glycemic control, SGLT-2 inhibitors work by blocking the SGLT-2 receptors in the proximal tubule of the kidney. This blockade prevents the reabsorption of glucose back into the body, and results in enhanced excretion of glucose in the urine [18,19]. The SGLT-2 receptors are highly concentrated early in the kidney tubule and are a high-capacity glucose transporter, accounting for > 90% of renal glucose reabsorption as compared with SGLT-1 receptors which are more populous in the GI tract [18,19]. Several proposed theories have sought to explain the cardiovascular benefits of SGLT-2 inhibitors, which appear to be independent of glucose lowering [13]. These potential mechanisms are multifactorial and include: (1) reduction in inflammation; (2) improvement in natriuresis/diuresis; and (3) promotion of the use of ketones as a secondary energy source [14].

Although the exact mechanism by which SGLT-2 inhibitors help reduce mortality and morbidity among heart failure patients remains elusive, results reported by many clinical trials established the clear cardiovascular benefits when SGLT-2 inhibitors are used among heart failure patients [18,19]. One theory suggests that these agents increase circulating ketones, inhibiting NLRP3, a protein known to contribute to chronic inflammation. The decrease in inflammation will subsequently prevent extracellular matrix remodeling and fibrosis [15]. Furthermore, SGLT-2 inhibitors promote osmosis and diuresis by inhibiting sodium reabsorption in the kidney’s proximal tubules [13]. Consequently, there is a reduction in blood pressure, cardiac afterload, and improved cardiac efficiency [14]. Lastly, during cardiac decompensation, there is a decrease in mitochondrial oxidation. As SGLT-2 inhibitors increase plasma ketone levels, this may serve as a critical energy source to supplement the failing heart [16]. Additional mechanisms that may contribute to the cardiovascular benefits of SGLT-2 inhibitors to a lesser degree include the reduction of plasma uric acid, promotion of cardiac autophagy, and improvement in erythropoiesis [15].

## 3. Guideline Recommendations for SGLT-2 Inhibitors Use among HF Patients

SGLT-2 inhibitors are the first class of antidiabetic agents to be granted FDA approval for the treatment of heart failure with reduced ejection fraction (HFrEF) [17,18]. The use of SGLT-2 inhibitors in patients with HF is recommended based on the 2022 AHA/ACC/HFSA Guideline for the Management of Heart Failure across all ranges of ejection fraction (EF) [4]. This includes a 1A recommendation in HFrEF and a 2a-BR recommendation (moderate strength of recommendation and quality of evidence) in HFmrEF and HFpEF [4].

Moreover, the use of SGLT-2 inhibitors with a proven heart failure benefit—empagliflozin, dapagliflozin, canagliflozin, and ertugliflozin—for the treatment of diabetes is recommended by The American Diabetes Association and The Kidney Disease: Improving Global Outcomes (KDIGO) guidelines among diabetic patients with confirmed HF or at high risk of HF [19,20].

## 4. Dapagliflozin

### 4.1. Approved Dose, Dose Adjustment, Adverse Events, and Monitoring

When used outside of antihyperglycemic management, the dose of dapagliflozin is 10 mg by mouth daily without regard to meals and without need for dose titration [4,17]. In patients with HFrEF for reduction of CV death and hospitalization for heart failure, there is sufficient data to support the initiation of therapy in eGFR ≥25 mL/min/1.73 m^2^ with continuation up to but excluding dialysis [4,17].

Adverse effect monitoring should include pharmacovigilance for hypotension secondary to hypovolemia and volume contraction, euglycemic ketoacidosis, acute kidney injury, genital mycotic infections (at risk individuals: elderly, female, uncontrolled hyperglycemia, or prior history), hypoglycemia when combined with insulin, Fournier gangrene, and pyelonephritis [21,22]. Routine urinalysis should not be monitored while on SGLT-2 inhibitor therapy if no symptoms of genital mycotic infections present. Aside from rare but serious side effects of Fournier which average a time of occurrence of 9 months (5–49 months), the majority of side effects are mild dose-dependent effects evident in the 1st month of use and do not typically require discontinuation [21,22]. There is a well-documented transient decrease in eGFR that occurs in the first 4 weeks of titration that recovers and stabilizes after initiation, thereafter showing renal protective benefits [21,22]. It is prudent to monitor patients on SGLT-2 inhibitors therapy for acute metabolic stress, dehydration, or prolonged fasting for the aforementioned reasons [21,22].

### 4.2. Efficacy Evidence behind the Use Dapagliflozin

The first observations of the benefits of SGLT-2 inhibitors on Heart Failure were noted in the EMPA-REG (2015), CANVAS trial (2017), and Declare-TIMI 58 (2018) cardiovascular outcomes trials (CVOTs) [23,24]. These initial three trials were conducted in diabetic patients diagnosed with ASCVD or at risk for ASCVD [23,24]. DECLARE-TIMI 58 failed to show a significant reduction of primary 3-point MACE in 17,160 diabetic patients (Average A1c 8.3%) with or without ASCVD [24]. Whereas the prior 2 CVOT required ASCVD for inclusion, DECLARE-TIMI 58 had only 41% of included patients with ASCVD [24]. However, it did generate interesting exploratory secondary endpoints: reduced CV death (HR 0.83 (0.73–0.95) *p* = 0.005) & reduced HF hospitalization (HR 0.73 (0.61–0.88)) [24]. There was also a notable >40% reduction in eGFR, new end-stage renal disease, or death from renal or cardiovascular causes (HR 0.76 (95% CI 0.67–0.87)) and a non-significant numerical reduction of death from any cause (HR 0.93 (95% CI 0.82–1.04)) [24].

The subsequent landmark trial DAPA-HF (2019) further validated dapagliflozin’s effects on HF and was the first of the SGLT-2 inhibitors trials in HF [25]. DAPA-HF studied dapagliflozin 10 mg once-daily versus placebo in 4744 patients NYHA II-IV HF with EF ≤40% and prespecified NT-proBNP thresholds for HHF or atrial fibrillation [25]. The primary composite of worsening HF or CV death was met with a HR 0.74 (0.65–0.85); *p* < 0.001 [25]. In a pre-specified sub-group analysis, the primary outcome was maintained irrespective of diabetes status, A1c range, or glucose-lowering effect [25]. What raised the greatest interest was the secondary finding that all-cause mortality was significantly reduced by HR 0.83 (0.71–0.97) [25]. Time to significant benefit was seen within 28 days (about 4 weeks) of starting medication, and the final number needed to treat was 43.5 to prevent one death [25].

The DELIVER trial published in late 2022 followed the AHA/ACC/HFSA 2022 guideline publication but corroborated the findings of EMPEROR-preserved in HFmrEF and HFpEF [26]. DELIVER (2022) included 6263 patients with NYHA II-IV, EF ≥ 40%, and eGFR ≥ 25 mL/min/1.73 m^2^ [26]. It showed a significant reduction in the composite of worsening HF (unplanned hospitalization or urgent visit for HF) or CV death with HR 0.82 (0.73–0.92); *p* < 0.001 [26]. These outcomes were similar across pre-specified subgroups including diabetes status and ejection fraction [26].

## 5. Empagliflozin

### 5.1. Approved Dose, Dose Adjustment, Adverse Events, and Monitoring

Unlike when it is used for hyperglycemia management, the empagliflozin HF FDA-approved dose is 10 mg by mouth daily [4,18]. Empagliflozin can be taken with or without food [18]. There is no recommended dose adjustment for empagliflozin when it is used for HF treatment [4,18]. However, studies evaluated the safety and efficacy of empagliflozin among HF patients only enrolled patients with eGFR of ≥20 mL/min/1.73 m^2^ [4,18]. Empagliflozin is currently contraindicated among hemodialysis patients [18].

Empagliflozin has several adverse effects to be monitored for, including hypotension, ketoacidosis, acute kidney injury, genital mycotic infections, hypoglycemia when combined with insulin, a rare but serious Fournier gangrene, and pyelonephritis [18]. Nevertheless, only urinary tract infections and female genital mycotic infections were reported among >5% of patients who used Empagliflozin [18]. Empagliflozin can result in ketoacidosis, particularly in patients with type 1 diabetes [18]. Thus, the use of empagliflozin for glycemia control among type 1 diabetic patients is not indicated [18]. Patients with predisposing factors to ketoacidosis should be closely monitored when empagliflozin is deemed necessary, including pancreatic disorders, history of pancreatitis, pancreatic surgery, and alcohol abuse [18]. There are no specific monitoring parameters when empagliflozin is used for HF [18]. It is only recommended that clinicians educate patients on the importance of hydration and urinary tract infection symptoms [18].

### 5.2. Efficacy Evidence behind the Use of Empagliflozin

EMPA-REG OUTCOME was the first randomized clinical trial to report a statistically significant lower death from cardiovascular causes, non-fatal myocardial infarction, or non-fatal stroke with empagliflozin among high-risk diabetic patients [23]. Additionally, empagliflozin resulted in a statistically significant reduction in heart failure hospitalization and heart failure composite outcome (hospitalization for heart failure or death from cardiovascular causes excluding fatal stroke) [23]. EMPA-REG OUTCOME included patients with type 2 diabetes and eGFR ≥30 mL/min/1.73 m^2^ [23]. Based on subgroup analysis, a statistically significant reduction in the composite primary outcomes (cardiovascular mortality, non-fatal myocardial infarction, or non-fatal stroke) was only found among elderly patients (≥65 years old), A1C < 8.5%, Asian race, BMI < 30, eGFR 60–90 mL/min/1.73 m^2^, urine albumin-to-creatinine ratio >300 mg/g, and not on insulin therapy while receiving empagliflozin [23]. Death from cardiovascular causes was similar between 10 and 25 mg of empagliflozin [23]. The EMPA-TROPISM clinical trial tested the hypothesis of empagliflozin cardiovascular benefit among nondiabetic HFrEF patients [27]. EMPA-TROPISM included heart failure patients with a left ventricular ejection fraction (LVEF) of <50% and stable symptoms and medical regimen for the last three months [27]. Among HFrEF nondiabetic patients in EMPA-TROPISM, empagliflozin 10 mg daily significantly improved left ventricular systolic function, functional capacity, and quality of life [27].

Two major landmark clinical trials, EMPEROR-Reduced and EMPEROR-Preserved, further investigated the role of empagliflozin among heart failure patients [28,29]. EMPEROR-Reduced included heart failure patients with a LVEF of ≤40% [28]. Approximately 50% of patients included had diabetes at baseline. Only empagliflozin 10 mg daily was used [28]. EMPEROR-Reduced concluded that in diabetic and nondiabetic patients with HFrEF, empagliflozin reduced cardiovascular mortality and hospitalization for the progression of HF [28]. EMPEROR-Preserved included heart failure patients with LVEF of >40% [29]. Similar to EMPEROR-Reduced, about 50% of patients included in EMPEROR-Preserved had diabetes at baseline [29]. The significant reduction of cardiovascular mortality and hospitalization for progression of HF irrespective of the diagnosis of diabetes was reported in EMPEROR-Preserved, similar to EMPEROR-Reduced [29]. Together, these studies showed a consistent reduction in cardiovascular mortality and hospitalization for the progression of HF regardless of the LVEF and diagnosis of diabetes [28,29]. 

EMPULSE is the largest clinical trial to date examining the use of empagliflozin initiation during an acute hospital admission for heart failure [30]. Over 50% of patients had an LVEF of ≤40%, and only 45% had diabetes at baseline [30]. Among patients with acute decompensated heart failure, empagliflozin administration was associated with a significant clinical benefit at 90 days, regardless of ejection fraction or diabetes status [30]. Moreover, the clinical benefit of empagliflozin was independent of symptomatic impairment at baseline upon randomization [30]. Furthermore, empagliflozin was associated with fewer deaths, improved quality of life, and reduced body weight without safety concerns [30]. 

## 6. Early versus Late Initiation of SGLT-2 Inhibitors in Clinical Practice

AHA/ACC/HFSA guidelines do not specify how best to perform therapy sequencing and when to initiate guideline-directed treatment [4]. However, the guidelines recommended early and “without delay” GDMT initiation [4]. Thus, GDMT sequencing and specific time of initiation remain controversial. However, evidence from clinical trials showed significant clinical benefits of SGLT-2 inhibitors with early initiation, within days to weeks after initiation [29,30].

Prior to the FDA approval of SGLT-2 inhibitors, the historical initiation of the therapy and sequencing focused on maximizing beta-blockers and renin-angiotensin-aldosterone system inhibitors (RAASi) to target doses before mineralocorticoid receptor antagonists (MRA) or SGLT-2 inhibitors initiation. This could ultimately result in unnecessary delays in initiating crucial GDMT therapies. Based on the landmark trials in HF, SGLT-2 inhibitors have proven efficacy independent of the presence or absence of other HFrEF therapies [30]. Moreover, SGLT-2 inhibitors have proven to be safe and, in some cases, improve the tolerability of other GDMT when combined [30]. Furthermore, 30-day cardiovascular benefit and in-hospital safety and tolerability upon initiation were found among patients using empagliflozin and dapagliflozin [30]. The cardiovascular benefit was seen as early as 18–28 days after initiation [30].

A major analysis of the DAPA-HF trial sustained the benefit of dapagliflozin over placebo whether patients were optimized on guideline-recommended doses or not [25]. Despite only 33% of patients in EMPULSE being on GDMT, empagliflozin was still found to be as safe and effective as was previously reported in DAPA-HF and EMPEROR-Reduced [25,28]. Hyperkalemia increases the risk of life-threatening arrhythmia, and two of the HF pillars (RAASi and MRA) can result in hyperkalemia [17,18]. The risk of hyperkalemia is even higher when there is a decline in kidney function [17,18]. SGLT-2 inhibitors have consistently been associated with reduced adverse kidney outcomes and slow the progression of CKD independent of diabetes status, and this effect was preserved in the presence or absence of ACEi and ARB [31,32]. Additionally, SGLT-2 inhibitors increase the electronegative charge in the tubular lumen that regulates potassium excretion in the distal nephron [17,18]. A meta-analysis of 49,875 patients showed that using SGLT-2 inhibitors reduced the risk of severe hyperkalemia among patients with type 2 diabetes without increasing the risk of hypokalemia [33]. Therefore, SGLT-2 inhibitors s may help the tolerability of other GDMT.

The cumulative evidence supports the initiation of all GDMT as early as possible, as their benefits were found to be independent of each other [4]. SGLT-2 inhibitors are no exception and continued to show cardiovascular benefit and tolerability among HF patients regardless of the presence or absence of other GDMT. Due to the lack of titration and overall tolerability with concomitant GDMT, the initiation of SGLT2i should be considered as early as possible, either in a simultaneous or rapid sequence fashion with other HF therapies. Based on the most recent guidelines and benefits shown in large major clinical trials, SGLT-2 inhibitors must be considered as essential therapy among heart failure patients, in conjunction with ARNi/ACEi/ARB, MRA, and beta-blockers [4]. Similar to all medications, risk versus benefit should be evaluated for each patient by a specialized provider. Clinicians should avoid use of SGLT-2 inhibitors among patients with type 1 diabetes and patients who are at a higher risk of lower extremity amputation (neuropathy, peripheral vascular disease, ulcers, history of previous amputation). Lastly, patients with severe volume depletion and worsening kidney function should be carefully evaluated before early initiation of SGLT-2 inhibitors.

## 7. The Role of the Pharmacist in the Early Initiation of Therapy and Adherence

Although medication dispensing represents a core role of hospital system pharmacists, modern medicine and clinical pharmacy have expanded the pharmacists’ roles and provided the opportunity for them to be an active part of the medical rounds at the bedside and to perform patient education [34,35]. This involvement allows for transitions from the retrospective medication review by pharmacists to providing their specialized knowledge and recommendations at the time of prescribing [34,35,36]. Additionally, pharmacists can help overcome clinical inertia by bringing provider level updates to rounding. Due to the rapidly increasing clinical complexity of medicine, medication errors can occur in any setting (inpatient, outpatient, or clinic) and during any stage of the patients care [34,35]. Pharmacists are pharmacotherapy experts, and their education and training provide the opportunity to help reduce medication errors [34,35].

A meta-analysis has found that pharmacists’ interventions at transitions of care estimated a 37% reduction in medication errors and a decrease in emergency department visits after hospital discharge [37]. Another randomized clinical trial has reported that partnering with pharmacists while caring for patients admitted to the general medical and emergency units resulted in a 75% reduction in medication errors [38]. Furthermore, a prospective study found that pharmacists’ involvement in cardiovascular disease management resulted in clinical and cost benefits [39]. Pharmacists in this setting can help overcome the commonly held prescribing barriers of cost, provider comfort, and safety/monitoring of SGLT-2 inhibitors [40,41]. As with all novel medications, cost is a concern for both health care providers and patients. Pharmacists overcome costs at transitions of care by connecting patients with manufacturer’s assistance and completing prior authorizations [40,41].

With the fast-growing evidence and as the use of SGLT-2 inhibitors becomes more popular in patients with or without diabetes, it is crucial to understand the benefits of these medications and to communicate them to the patient and health care providers [41,42]. Pharmacists can play a major role in providing such education. Additionally, most patients with diabetes also have other cardiovascular disease or HF. Clinical pharmacists can help optimize the patient’s regimen and lower the pill burden by providing a medication that targets and benefits multiple disease states [41,42]. Moreover, clinical pharmacists can counsel patients and provide reasonable expectations on potential side effects when patients are started on these medications [41,42]. There are no specific monitoring parameters upon the initiation of SGLT-2 inhibitors. However, patients are at risk of genital infection and over-diuresis with SGLT-2 inhibitors therapy. Thus, patient education on hydration and urinary tract infection symptoms is encouraged [18].

Cardiovascular diseases such as heart failure are the leading cause of death in the United States and worldwide, and their management costs more than two-thirds of global healthcare expenditure [40,41]. Thus, pharmacists’ involvement as pharmacotherapy experts on heart failure teams may assist medical professionals in safely initiating SGLT-2 inhibitors early [40,41,42]. Having pharmacists involved in caring and planning for heart failure patients has been shown to reduce all-cause and HF-related hospitalizations and improve overall patient health and satisfaction [43]. Additionally, studies have shown higher adherence to HF guidelines-directed therapies when physicians collaborated with pharmacists [43,44]. Furthermore, the IMPLEMENT-HF pilot study showed that pharmacists involvement in virtual GDMT optimization team was associated with improved heart failure therapeutic titration [45]. Lastly, patients’ adherence to their HF guidelines-directed therapies was significantly higher when pharmacists were involved in their hospital care [46]. Common barriers of initiation and optimal patient characteristics are summarized in Table 1.

## 8. Limitations

This was a review article and included experts’ opinions. Any review article comes with the risk of potential bias and is subject to systematic and random errors. Due to the nature of this study, no statistical analysis was performed. Moreover, we only discussed SGLT-2 inhibitors currently approved for HF in the US. To date, no randomized clinical trial has evaluated the safety and efficacy of empagliflozin versus dapagliflozin among HF patients. Thus, we could not collate and contrast empagliflozin and dapagliflozin in clinical practice.

## 9. Conclusions

Evidence-based practices for the management of HF patients are rapidly growing. SGLT-2 inhibitors are currently approved by the FDA for treating HFrEF and demonstrated morbidity and mortality benefits within 30 days of initiation. The exact mechanism by which SGLT-2 inhibitors attenuate the inflammatory process remains unknown. SGLT-2 inhibitors were found to be safe and effective even with early initiation and alongside other concomitant GDMT. Pharmacists play a major role in patients’ selection, early initiation, and adherence to HF guidelines-directed medical therapies, including SGLT-2 inhibitors.

## Figures and Tables

**Table 1:** Safe Initiation Criteria and Ideal Patients for HFrEF Treatment Pillar.

	ACEi/ARB/ARNi	Beta-Blockers	MRA	SGLT-2 Inhibitors
Safe initiation criteria	Hemodynamically stable (SBP > 100 mm Hg) Not currently on vasopressors or inotropic support eGFR > 30 mL/min/1.73 m^2^ K < 5 mEq/L	Hemodynamically stable (SBP > 100 mm Hg)	Hemodynamically stable (SBP > 100 mm Hg)	Hemodynamically stable (SBP > 100 mm Hg) eGFR > 30 mL/min/1.73 m^2^ when dapagliflozin is used and > 25 mL/min/1.73 m^2^ when empagliflozin is used. Euvolemia or hypervolemia
Contraindication	History of angioedema The use of ACEi in the past 36 h of ARNi initiation Severe aortic stenosis Pregnancy	HR < 50 BPM Patients with volume overload	K ≥ 5.5 mEq/L SCr > 2.5 mg/dL in men and > 2 mg/dL in women	Type 1 diabetes Hemodialysis
Ideal patients	Type 1 and 2 diabetes Patients with chronic kidney disease	Patients with other cardiovascular diseases	Patients with other cardiovascular diseases Hypotension limiting the initiation or optimization of GDMT. Patients diagnosed with HFpEF	Type 2 diabetes Patients with chronic kidney disease Hypotension limiting the initiation or optimization of GDMT.

ACEi: Angiotensin-converting-enzyme inhibitors; ARB: Angiotensin receptor blockers; ARNi: Angiotensin receptor/neprilysin inhibitor; MRA: Mineralocorticoid receptor antagonists; GDMT: Guidelines-directed medical therapy; HR: Heart rate; BPM: Beat per minute; eGFR: Estimated glomerular filtration rate; SCr: Serum creatinine; SBP: Systolic blood pressure.

## Data Availability

Not applicable.
